# Highly Transparent Aromatic Polyamides from Unsymmetrical Diamine with Trifluoromethyl Groups

**DOI:** 10.3390/polym14030501

**Published:** 2022-01-27

**Authors:** Seong Jong Kim, Inah Kang, Taejoon Byun, Jongho So, Sang Youl Kim

**Affiliations:** Department of Chemistry, Korea Advanced Institute of Science and Technology (KAIST), Daejeon 34141, Korea; krisj@kaist.ac.kr (S.J.K.); gk0784@kaist.ac.kr (I.K.); ruminaire@naver.com (T.B.); jonghoso@kaist.ac.kr (J.S.)

**Keywords:** high temperature polymer, polyamides, trifluoromethyl group

## Abstract

Soluble and transparent wholly aromatic polyamides (PAs) were synthesized from an unsymmetrical diamine monomer having trifluoromethyl (CF_3_) groups, 4-(4′-aminophenoxy)-3,5-bis(trifluoromethyl)aniline. The monomer was polymerized with several dicarboxylic acid monomers via the Yamazaki–Higashi polycondensation method. All of the synthesized polyamides have an amorphous morphology, and they are soluble in many polar organic solvents at room temperature. Flexible and transparent films of the polyamides were prepared by solution casting and these polymer films show good optical transparencies with cut-off wavelengths of 337–367 nm and transparencies of 88%–90% at 550 nm. In addition, all the polymers were thermally stable over 400 °C and exhibited glass transition temperatures (*T*_g_) higher than 300 °C. Unsymmetrically inserted trifluoromethyl groups on polyamides improves the solubility as well as the transparency of the polymers while maintaining good thermal properties. They also showed low refractive indices around 1.5333~1.5833 at 633 nm owing to the existence of low polarizable trifluoromethyl groups.

## 1. Introduction

The strong demand for portable/wearable electronic devices with various form factors requires the development of unprecedented display devices as a key element for the realization of the next-generation devices, and transparent polymeric materials are receiving great attention because they can replace rigid glass substrate for transparent and flexible display [1,2]. Unfortunately, commonly used transparent polymeric materials, including poly(ethylene terephthalate) (PET), poly(ethylene naphthalate) (PEN) and polycarbonate (PC), are found to be inappropriate to use as substrate materials for flexible organic light-emitting diode (OLED) displays mainly because it cannot withstand the fabrication conditions currently employed for thin film transistor (TFT) manufacturing [3,4]. For instance, the direct fabrication of transparent and flexible oxide thin-film transistors onto a polymer substrate often requires post thermal annealing and the polymer films should maintain its integrity during the heating process above 300 °C [5,6,7]. However, the glass transition and melting temperatures (*T*_g_, and *T*_m_) of the aforementioned polymer films are not high enough for TFT fabrication process [8].

Among the many engineering plastics, aromatic polyimides (PIs) are extensively studied as a promising candidate for flexible substrates owing to its excellent thermo-oxidative stability together with exceptionally high *T*_g_ that meets the requirements for device production [9,10]. Despite the outstanding performance of PIs, these polymers are difficult to process because of its high melting temperature and poor solubility. Moreover, PI films often show an intense color because of their rigid, highly conjugated aromatic structure and inherent capability to form a so-called intermolecular charge-transfer (CT) complex between electron-deficient dianhydride and electron-rich diamine units [11,12]. To overcome these problems, various structural modifications have been attempted on PIs [10,13,14,15,16,17,18].

Aromatic polyamides (PAs) are another representative class of high performance polymeric materials and they are well known for high thermal resistance and transition temperature comparable to PIs. Their outstanding properties arise from strong and highly directional interchain hydrogen bonds which grant high cohesive energy between the polymer chains [19,20]. However, the application of PAs in the form of films or membranes often get frustrated because of the rigid aromatic chains interacting strongly with multiple hydrogen bonds which make them intractable and insoluble in organic solvents. Therefore, in order to expand their application span, much research effort has been devoted to finding synthetic strategies for preparing soluble and processable PAs while retaining outstanding physical properties. Several effective approaches including the incorporation of flexible or asymmetric linkages, the attachment of bulky substituents onto the main chains and the copolymerization of monomers having nonplanar structures have been reported over the last few decades [21,22,23,24,25,26,27].

Among the various approaches, the insertion of CF_3_ groups onto polymer main chains is found to be effective in making soluble PAs without losing their outstanding thermal properties, not only because bulky trifluoromethyl groups effectively prevent the packing of the polymer main chains, but also because of the strong bond strength of the C-F bond [28,29,30,31,32].

Particularly, soluble fluorinated PAs could have their own advantage over PIs in terms of coloration because CT interaction could be more alleviated through the replacement of a highly electro-deficient dianhydride unit into diacids. For instance, we recently reported the transparent poly(amide-imide)s, which were synthesized from 2,2′-bis(trifluoromethyl)benzidine and trimellitic anhydride [33]. By changing some of the imide functional groups to amide groups in polyimide main chains, the resulting polymers had better solubility and optical properties compared to PIs from the same diamine and pyromellitic dianhydride [34], while maintaining good thermal properties. Therefore, fluorinated PAs can also be a good platform for achieving transparent polymeric substrate with high thermal stability. However, despite the potentials of PAs, the majority of research efforts have dealt with PIs and only a few examples of optically transparent PAs have been investigated thus far [35,36,37,38,39].

In this study, we prepared a new series of fluorinated PAs from unsymmetrical diamine monomer, 4-(4′-aminophenoxy)-3,5-bis(trifluoromethyl)aniline, which contains trifluoromethyl groups on 2, 6-positions of 4,4′-oxydianline. This monomer was first developed in our previous research on fluorinated PIs and the synthesized polymers showed remarkably enhanced solubility in solvents together with reduced coloration while retaining excellent thermal properties of PIs [40]. Therefore, we expect that the PAs prepared from the same monomer exhibit not only good organosolubility and high thermal stability, but also improved optical clarity compared to the analogous PIs as the intermolecular CT interactions could be mitigated by the replacement of a dianhydride unit with diamide.

## 2. Materials and Methods

### 2.1. Materials

The compound **1**, 2-bromo-5-nitro-1,3-bis(trifluoromethyl)benzene, and **3**, 4-(4′-aminophenoxy)-3,5-bis(trifluoromethyl) aniline, were prepared as reported previously [40]. Terephthalic acid and isophthalic acid were purchased from TCI (Tokyo Chemical Industry Co. Ltd., Tokyo, Japan) and purified by vacuum sublimation. 4,4′-Dicarboxydiphenyl ether and 2,2-bis(4-carboxyphenyl)hexafluoropropane were also purchased from TCI and purified by recrystallization in ethanol and acetonitrile, respectively. Anhydrous *N*-methyl-2-pyrrolidone (NMP) and anhydrous pyridine were purchased from Sigma-Aldrich (Burlington, MA, USA) and were used without purification. Calcium chloride (CaCl_2_) was obtained from Junsei (Tokyo, Japan) and was dried under vacuum at 180 °C overnight prior to use. All other commercially available chemicals (reagent-grade) were used without purification.

### 2.2. Measurements

The nuclear magnetic resonance (NMR) spectra of the compounds were taken on a Bruker Fourier Transform Avance 400 spectrometer (Bruker, MA, USA). The NMR chemical shift was described in ppm (parts per million) with tetramethylsilane as a reference. Patterns of the peaks were noted as s (singlet), d (doublet), dd (doublets of doublet), t (triplet), q (quartet), or m (multiplet). The FT-IR (Fourier-transform infrared) spectra of the compounds were taken with a Bruker EQUINOX-55 spectrophotometer (Bruker, Billerica, MA, USA). The FlashEA 1112 elemental analyzer (Thermo Fisher, Waltham, MA, USA) was used for elemental analysis (EA) of the synthesized compounds. The measurement of inherent viscosities of the polymers was carried out with an Ubbelohde viscometer (Witeg, Wertheim, Germany). Size exclusion chromatography (SEC) diagrams were recorded with a Viscotek TDA302 model (Malvern, Worcestershire, UK) having a packing column (PLgel 10 μm MIXED-B) at 35 °C, and tetrahydrofuran (THF) was used as an eluent. The number average (M_n_) and weight average (M_w_) molecular weight of the polymers were obtained by using polystyrene standards. Thermal analyses (TGA and DSC) were carried out with a TA Instruments TGA Q50 and a DSC Q20 instrument (TA Instruments, New Castle, DE, USA) at a scanning rate of 10 °C/min under a nitrogen atmosphere, respectively. *T*_g_ (glass transition temperature) was measured from the second heating scan after cooling to 0 °C from 350 °C. Linear coefficients of thermal expansion (in-plane CTE) of the polymer films were obtained by a thermomechanical analyzer (TMA) using a TA TMA-Q400 (TA Instruments, New Castle, DE, USA). The measurements were performed 3 times in a heating range up to 300 °C at a rate of 5 °C/min, and CTE value was evaluated as an average rate of change in the temperature from 50 °C to 250 °C in the second and third heating runs. UV-visible spectrum was taken with a Shimadzu UV-2600 spectrometer (Shimadzu, Kyoto, Japan) in transmittance mode. The refractive index for transverse electric (TE, *n*_TE_) and transverse magnetic (TM, *n*_TM_) modes of the polymer films were obtained by using a Sairon SPA-4000 prism coupler (Sairon Technology, Gwangju, Korea) having a gadolinium gallium garnet (GGG) prism at 633 nm and 1310 nm at RT. The birefringence values (Δ*n*) were calculated from the difference of *n*_TE_ and *n*_TM_. Wide-angle X-ray diffraction (WAXD) measurements were carried out at RT on Rigaku SMARTLAB X-ray diffractometer (Rigaku, Tokyo, Japan) with a Cu Kα_1_ incident beam.

### 2.3. Model Reaction

Model Compound (**4**). A round-bottom flask (25 mL) with a three-necked inlet was charged with 3 (1.008 g, 3 mmol), benzoic acid (0.733 g, 6 mmol), NMP (13.5 mL), pyridine (3 mL), triphenyl phosphite (3.6 mL), and CaCl2 (0.3 g) in nitrogen atmosphere. The temperature of the reaction mixture was raised to 100 °C and the mixture was stirred for 3 h. During the reaction, small aliquots were taken from the mixture periodically and subjected to ^1^H NMR analysis for monitoring the conversion of reaction. After completion, the reaction mixture was cooled down to RT and extracted with ethyl acetate and water. The non-aqueous layer was dried under MgSO_4_ and then evaporated by using a rotary evaporator. The crude product was purified by passing through a silica column with ethyl acetate/hexane (*v*/*v* = 1:1.5) mixture as an eluent to give a white solid **4** (1.57 g, 96.12% yield). m.p. 212 °C. FT-IR (cm^−1^): 3440, 3320 (amide N–H); 1682, 1654 (amide C=O); 1270 (C–O–C); 1189–1156 (C–F in CF_3_). ^1^H NMR (DMSO-*d*_6_, 400 MHz, 25 °C, ppm): 10.86 (s, 1H), 10.24 (s, 1H), 8.04–7.99 (m, 2H), 7.95–7.90 (m, 2H), 7.72–7.48 (m, 8H), 6.82 (d, *J* = 4.54 Hz, 2H). ^13^C NMR (DMSO-*d*_6_, 100 MHz, 25 °C, ppm): 166.13, 165.28, 155.39, 144.06, 137.56, 134.90, 134.00, 133.87, 132.27, 131.45, 128.59, 128.33, 127.75, 127.55, 125.06 (q, *J* = 31.29 Hz), 122.59 (q, *J* = 5.45 Hz), 122.39 (q, *J* = 272.05 Hz), 114.94. EA calcd. for C_28_H_18_F_6_N_2_O_3_: C, 61.77%; H, 3.33%; N, 5.15%. Found: C, 60.77%; H, 3.20%; N, 4.96%.

### 2.4. Polymerization

**PA-1**. A mechanical stirrer and nitrogen inlet were installed on a 25 mL three-neck round-bottom flask, and then stoichiometric amount of diamine 3 (0.3362 g, 1 mmol) and terephthalic acid (0.1661 g, 1 mmol), NMP (4 mL), pyridine (1 mL), triphenylphosphite (1.2 mL), and CaCl_2_ (0.3 g) were charged into the flask. The reaction mixture was heated to 100 °C and was stirred for 8 h under argon atmosphere. After the polymerization reaction was complete, the viscous polymer solution was poured into vigorously stirred methanol. The precipitated polymer was filtered and washed thoroughly with methanol/water, then dried overnight in a vacuum oven at 100 °C. (0.4643 g, 99.5% yield). FT-IR (cm^−1^): 3305 (amide N–H); 3065 (aromatic C–H); 1662 (amide C=O); 1610-1475 (aromatic C=C); 1259 (C–O–C); 1199–1142 (C–F). ^1^H NMR (DMSO-*d*_6_, 400 MHz, 25 °C, ppm): 11.04 (s, amide), 11.00 (s, amide), 10.40 (s, amide), 10.35 (s, amide), 8.68 (s, 2H), 8.21 (s), 8.14 (s), 8.06 (s), 7.72 (s, 2H), 6.85(s, 2H). EA calcd. for C_22_H_14_F_6_N_2_O_3_: C, 56.66%; H, 2.59%; N, 6.01%. Found: C, 54.78%; H, 2.77%; N, 5.78%.

**PA-2**. The same procedure employed for **PA-1** was repeated with 1 mmol of isophthalic acid. (0.4652 g, 99.8% yield). FT-IR (cm^−1^): 3309 (amide N–H); 3068 (aromatic C–H); 1662 (amide C=O); 1610–1475 (aromatic C=C); 1236 (C–O–C); 1199–1142 (C–F). ^1^H NMR (DMSO-*d*_6_, 400 MHz, 25 °C, ppm): 11.08 (s, amide), 11.03 (s, amide), 10.43 (s, amide), 10.37 (s, amide), 8.69 (s, 2H), 8.67 (s), 8.59 (s), 8.49 (s), 8.28 (d, *J* = 7.60 Hz), 8.19 (d, *J* = 7.76 Hz), 8.11 (d, *J* = 7.6 Hz), 7.85-7.62 (m, 3H), 6.89-6.80 (m, 2H). EA calcd. for C_22_H_14_F_6_N_2_O_3_: C, 56.66%; H, 2.59%; N, 6.01%. Found: C, 55.43%; H, 2.77%; N, 5.83%.

**PA-3**. The same procedure used for **PA-1** was repeated with 1 mmol of 4,4′-dicarboxydiphenyl ether. (0.5582 g, 99.9% yield). FT-IR (cm^−1^): 3311 (amide N–H); 3068 (aromatic C–H); 1658 (amide C=O); 1597–1475 (aromatic C=C); 1244 (C–O–C); 1199–1144 (C–F). ^1^H NMR (DMSO-*d*_6_, 400 MHz, 25 °C, ppm): 10.86 (s, amide), 10.85 (s, amide), 10.22 (s, amide), 10.20 (s, amide), 8.65 (s, 2H), 8.11 (t, *J* = 8.46 Hz, 1H), 8.03 (t, *J* = 8.7 Hz, 1H), 7.69 (d, *J* = 7.32 Hz, 2H), 7.34-7.14 (m, 4H), 6.82 (d, *J* = 7.32 Hz, 2H). EA calcd. for C_28_H_16_F_6_N_2_O_4_: C, 60.22%; H, 2.89%; N, 5.02%. Found: C, 58.50%; H, 3.00%; N, 4.73%.

**PA-4**. The same procedure used for **PA-1** was taken with 1 mmol of 2,2-bis(4-carboxyphenyl)hexafluoropropane and 5 mL of NMP. (0.6790 g, 98.0% yield). FT-IR (cm^−1^): 3311 (amide N–H); 3068 (aromatic C–H); 1666 (amide C=O); 1257 (C–O–C); 1207–1142 (C–F). ^1^H NMR (DMSO-*d*_6_, 400 MHz, 80 °C, ppm): 10.87 (s, amide, 1H), 10.19 (s, amide, 1H), 8.62 (s, 2H), 8.12 (t, *J* = 7.36 Hz, 1H), 8.05 (t, *J* = 7.52 Hz, 1H), 7.71 (d, *J* = 7.68 Hz, 1H), 7.59 (t, *J* = 6.92 Hz, 1H), 7.52 (t, *J* = 6.94 Hz, 1H), 6.82 (d, *J* = 7.88 Hz, 2H). EA calcd. for C_31_H_16_F_12_N_2_O_3_: C, 53.77%; H, 2.33%; N, 4.05%. Found: C, 52.77%; H, 2.46%; N, 3.84%.

### 2.5. Preparation of Poly(Amide-Imide) Films

The polymers (0.1 g) were dissolved in 5 mL of DMAc at RT. The homogeneous solutions were passed through a 5.0 μm PTFE syringe filter and drop casted on a glass plate (5 × 5). The solvent was slowly evaporated in a vacuum oven at 60 °C for 2 h, and 180 °C overnight to remove residual solvents.

## 3. Results and Discussion

### 3.1. Monomer Synthesis

**Scheme 1:**
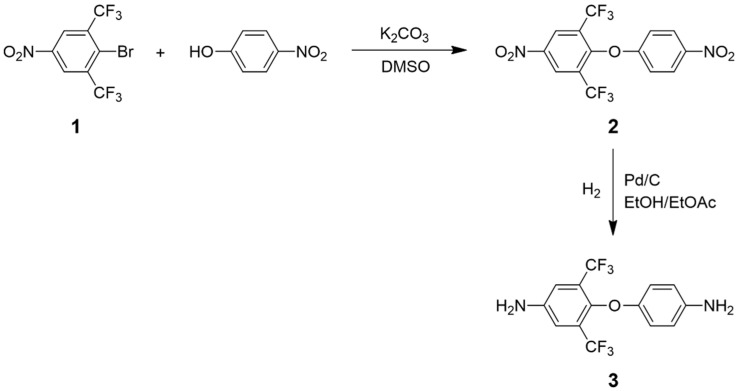
Synthesis of the diamine monomer having unsymmetrically substituted trifluoromethyl groups.

The diamine monomer with unsymmetrically substituted trifluoromethyl groups, 4-(4′-aminophenoxy)-3,5-bis(trifluoromethyl) aniline (**3**), was synthesized according to the previously reported procedure (Scheme 1) [40]. First, 4-nitrophenol was reacted with 2-bromo-5-nitro-1,3-bis(trifluoromethyl)benzene (**1**) through a nucleophilic aromatic substitution reaction (S_N_Ar) with K_2_CO_3_ as a base to make the dinitro compound **2**. Then, the dinitro compound was transformed to the diamine monomer **3** by Pd catalyzed hydrogenation reaction with the Pd/C catalyst.

### 3.2. Model Reaction

**Scheme 2:**
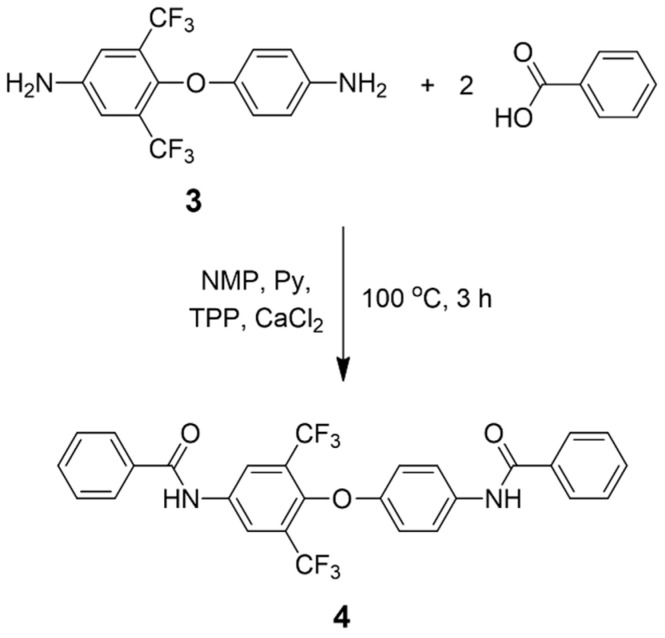
Model reaction of the monomer **3** with phthalic acid.

To investigate the reactivity of the diamine monomer for a condensation reaction, a model reaction was carried out in the polymerization condition. The model reaction was also conducted to make a model compound as a reference compound for analysis of chemical structures. The diamine monomer **3** was reacted with twice the amount of phthalic acid (Scheme 2), and the reaction progress was followed by ^1^H NMR. Between two amine groups of the monomer, the electron-rich amine was fully converted to amide in less than 30 min, whereas the electron-deficient amine exhibited less nucleophilicity and was fully consumed after 2 h. Even though the two amine groups of the monomer **3** have different electron densities, they have sufficient reactivity for the condensation reaction with phthalic acid, and the diamide model compound **4** was prepared quantitatively.

**Figure 1:**
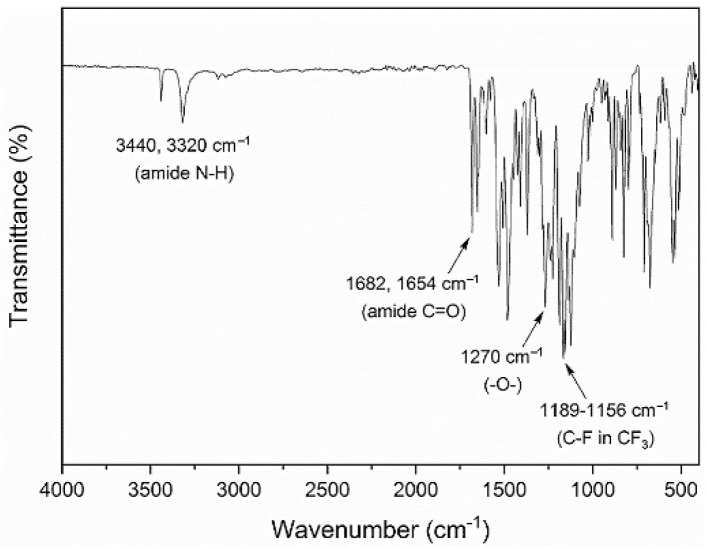
FT-IR spectrum of model compound **4**.

**Figure 2:**
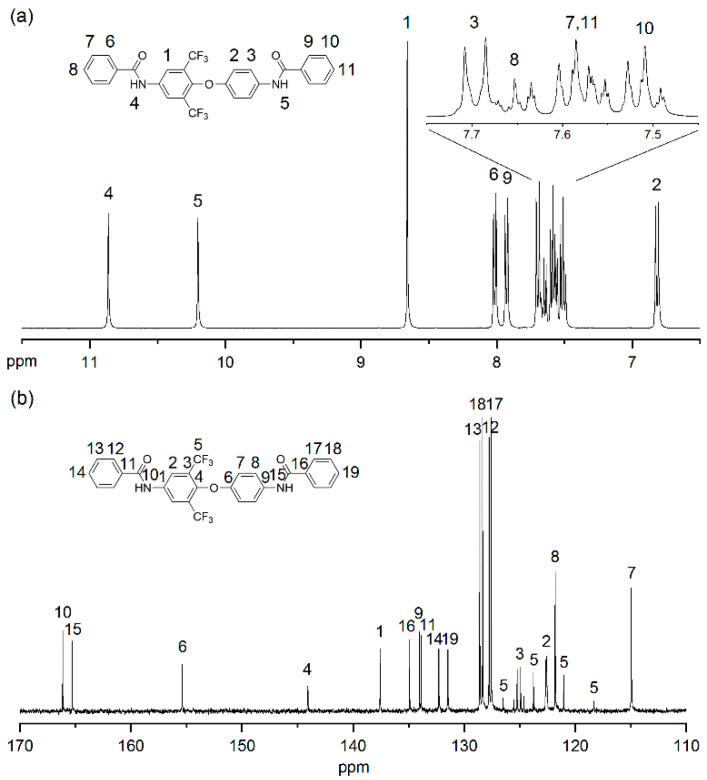
(**a**) ^1^H and (**b**) ^13^C NMR spectrum of the model compound **4** (DMSO-d_6_).

The chemical structure of **4** was characterized by FT-IR, ^1^H and ^13^C NMR spectroscopy. The FT-IR spectrum of the model compound exhibited characteristic bands at 3440, 3320 cm^−1^ and 1682, 1654 cm^−1^ corresponding to N-H and C = O stretching of amide, respectively (Figure 1). The ^1^H and ^13^C NMR spectra indicated the successful synthesis of the diamide model compound (Figure 2). Due to the asymmetric presence of trifluoromethyl groups on the compound, the peaks of proton and carbon on each phthalamide unit were observed at the different position of chemical shifts in the NMR spectra, where the peaks of phthalamide attached to the phenyl ring with trifluoromethyl groups moved to further downfield because of the electron withdrawing nature of the trifluoromethyl groups. The good agreement of spectroscopic data clearly supported the predicted structure.

### 3.3. Polymerization

**Scheme 3:**
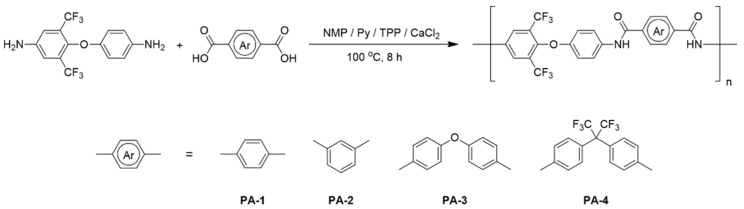
Polymerization of **3** with aromatic dicarboxylic acids.

With the successful result of the model reaction, a series of polyamides (PAs) were prepared from **3** and commercially available aromatic dicarboxylic acids via the Yamazaki–Higashi polycondensation method [41], as depicted in Scheme 3. The diamine monomer **3** was polymerized with the same amounts (stoichiometric) of diacid monomers in NMP at an initial content of solid around 10 *w/v* %. The polymerization was performed at 100 °C for 8 h and the polymerization reaction proceeded without premature precipitation of the polymeric product. After the polymerization reaction was complete, white fibrous polymers were produced by precipitating the polymer solutions in methanol and the polymer product was obtained quantitatively.

The inherent viscosities, molecular weight, and the results of the elemental analysis of the PAs are summarized in Table 1. The PAs have inherent viscosities of 0.80–1.28 dL/g in DMAc at 30 °C. PA1 and PA2 show different viscosity values even though they have a similar chemical structure and average molecular weight. It seems that the viscosity difference stems from the connectivity difference. The contour length of PA-1 (para-connected) is larger than PA-2 (meta-connected). In addition, because all PAs were soluble in THF, the molecular weight could be measured by THF-GPC (Figure S1). The number average (*M*_n_) and weight-average (*M*_w_) molecular weights of the PAs were in the range of 45.4–61.8 kDa and 119.0–128.4 kDa, respectively, using polystyrene standards. The average molecular weights of PAs were large enough to make flexible as well as tough polymer films by solution-casting from the dimethylacetamide solutions.

**Figure 3:**
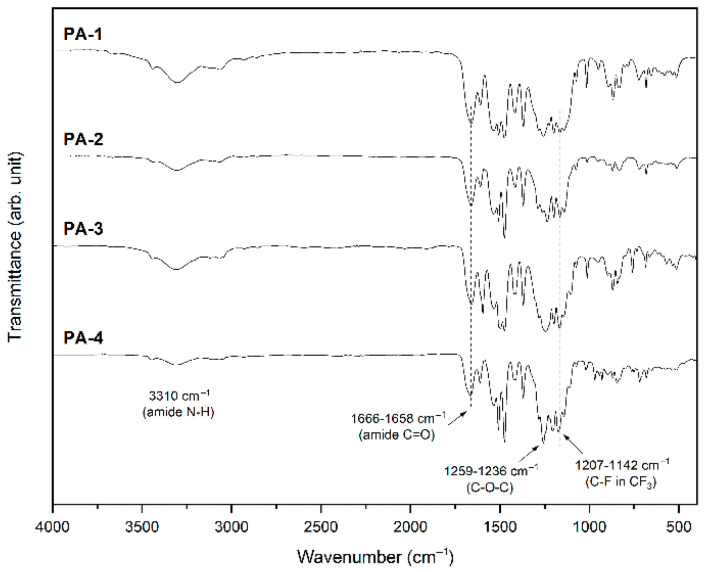
FT-IR spectra of polyamides (film).

**Figure 4:**
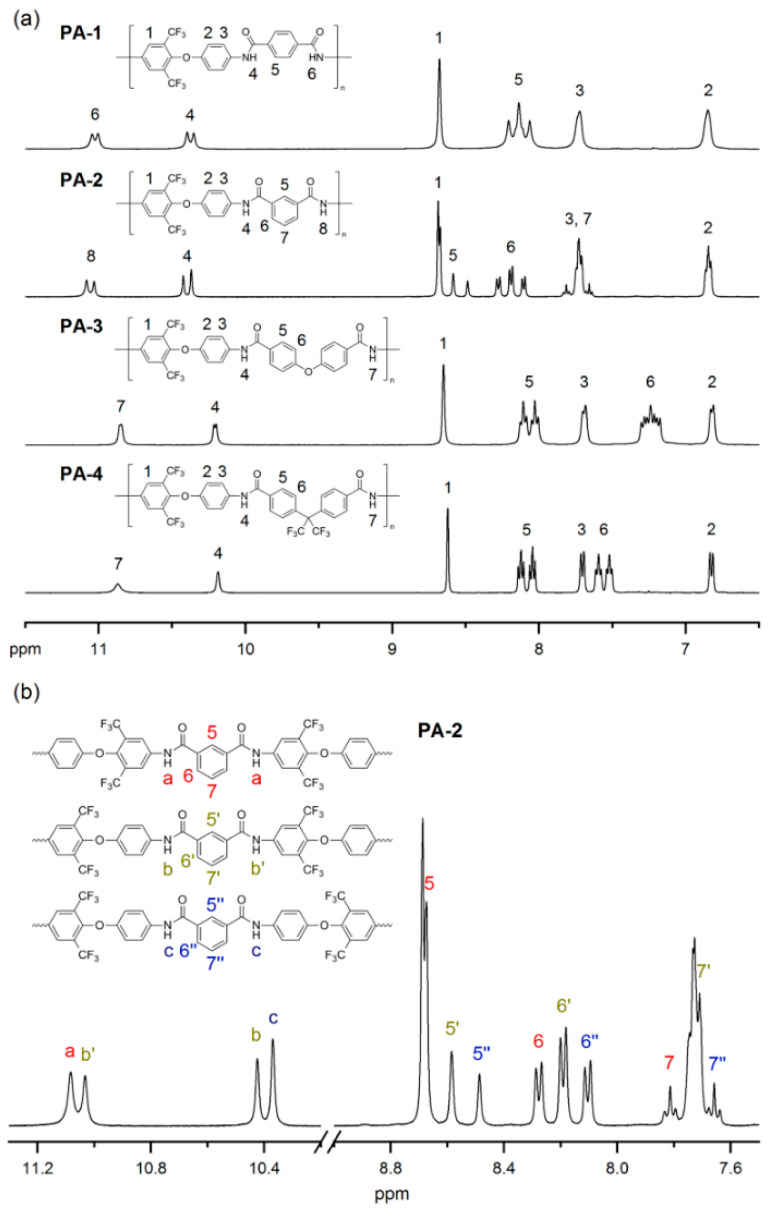
(**a**) ^1^H NMR spectra of **PA-1**, **PA-2**, **PA-3** (DMSO-*d*_6_, 25 °C) and **PA-4** (DMSO-*d*_6_, 80 °C). (**b**) magnified ^1^H NMR spectra of **PA-2**.

The chemical structures of the PAs were confirmed by FT-IR, ^13^C NMR (Figures S2–S5) and ^1^H NMR. The FT-IR spectra of PAs are presented in Figure 3. All PAs exhibited characteristic absorption bands corresponding to their functional groups around 3310 cm^−1^ (amide N-H), 1666–1658 cm^−1^ (amide C = O), 1259–1236 cm^−1^ (C-O-C), and in the range of 1207–1142 cm^−1^ due to carbon-fluorine stretching in CF_3_ groups. Figure 4a is the ^1^H NMR spectra of the PAs. The peaks of protons were well assigned to their predicted structure. Notably, the diacid proton peaks were divided and appeared at different positions owing to the asymmetric character of the diamine monomer unit in the PA chains. ^1^H NMR spectrum of **PA-2** is magnified in Figure 4b for closer inspection. As illustrated, the polymer chain consists of three different conformations (tail-to-tail, tail-to-head, head-to-head) derived from the asymmetric structure of **3**, and the protons from the diacid unit in each configuration exhibit different resonance peaks depending on its distance to the CF_3_ groups. The proton closer to CF_3_ groups appeared in more downfield region because of the electron withdrawing nature of trifluoromethyl group. The integral ratio of H_6_:H_6′_:H_6′_’ in **PA-2** spectrum was found to be 1:1.5:1, which deviates from the ideal ratio of 1:2:1 expected for polymers with statistical distribution. This difference strongly suggests that the reactivity of each amine group in **3** is unequal, which is consistent with the model reaction result. Despite the reactivity difference, the amine groups possessed enough reactivity to produce polymers with high molecular weights and thus no discernible end groups were detected in ^1^H NMR.

### 3.4. Polymer Properties

The solubility of PAs prepared in this study is shown in Table 2. All PAs were soluble in NMP (*N*-methyl-2-pyrrolidone), DMAc (*N*,*N*-dimethylacetamide), DMF (*N*,*N*-dimethylformamide), *m*-cresol, and tetrahydrofuran at RT without the aid of inorganic salts like LiCl or CaCl_2_. In addition **PA-4,** which contains a hexafluoroisopropylidene (-C(CF_3_)_2_) bridge on its main chain, showed better solubility and is even soluble in acetone. When compared with the PAs derived from 4,4′-oxydianiline (ODA) and 2-trifluoromethyl-4,4′-diaminodiphenylether [23,42,43], the PAs prepared here exhibited improved solubility. The solubility enhancement is ascribed not only to the bulky trifluoromethyl groups on the polymer main chains but also to the irregular chain structure stemming from the unsymmetrical monomer which prevents a close chain packing and reduces the interactions among polymer chains.

**Figure 5:**
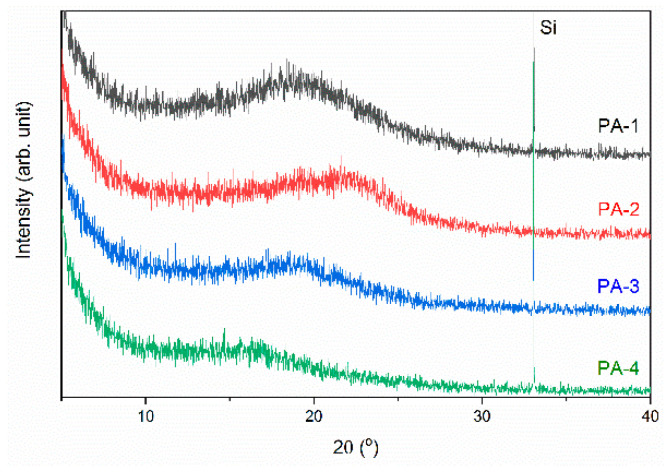
WAXD patterns of the PA films.

The effect of CF_3_ groups on the solution behavior of PAs in terms of crystallinity was further investigated by wide-angle X-ray diffraction (WAXD). As shown in Figure 5, WAXD patterns of the PAs only exhibited a broad plateau around 10–25° without a discernible diffraction peak, indicating that the synthesized PAs possess a highly amorphous character. This result was also in sharp contrast to that of ODA-based polyamides which exhibits sharp diffraction peaks on both *para*- and *meta*-linked polymers [42]. The two bulky CF3 groups on the diamine effectively prevent the close packing of polymer chains, which allows the easy diffusion of solvent molecules into the polymers.

**Figure 6:**
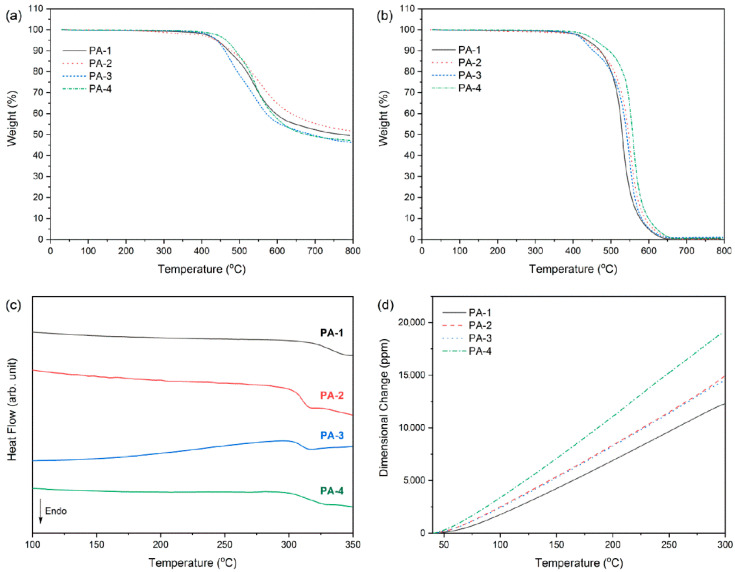
TGA curves of the PAs in (**a**) N_2_ and (**b**) air, (**c**) DSC curves (2nd heating run) and (**d**) TMA curves (3rd run) of the PAs.

The thermal properties of the PAs were investigated with TGA, DSC and TMA, and the results are shown in Table 3. The TGA result shows that all PAs possessed good thermal stability and were stable over 400 °C as shown in Figure 6a,b. The temperatures at which a loss of 5% weight (*T*_d5_) occurred were observed to be 437–465 °C in nitrogen and 434–458 °C in air, respectively.

Glass transition behaviors of the PAs were investigated by DSC experiment. Figure 6c shows the DSC thermogram obtained during the second heating cycle. All of the synthesized PAs showed a glass transition temperature (*T*_g_) over 300 °C and the most rigid **PA-1** possessed the highest *T*_g_ value of 334 °C. It is noteworthy that meta-linked **PA-2** exhibited higher *T*_g_ (310 °C) compared to the ODA-based polyamide (266 °C) [42], even though it exhibited a lower degree of chain packing in the WAXD measurement. The increment of *T*_g_ is ascribed to the presence of two CF_3_ groups at the ortho-positions of ether linkage, which hinders free rotation around the aryl ether bond and thus increases the stiffness of the polymer chain [40,44].

Thermo-dimensional stability of the polyamide films was studied by three consecutive TMA measurements (Figure 6d). The CTE (linear coefficients of thermal expansion) of the PA film samples were in the range of 49.7–76.7 ppm/°C. It has been known that polymers with more rectilinear structure exhibit a smaller CTE value [45], and the PAs described here follow this general structure–property relationship as well. The most rigid **PA-1** shows the lowest, the most twisted **PA-4** shows the highest, and the others show intermediate CTE values. In addition, the PAs maintain their thermo-dimensional stability up to 300 °C which is evidenced by their uniform CTE values between 2nd and 3rd runs.

**Figure 7:**
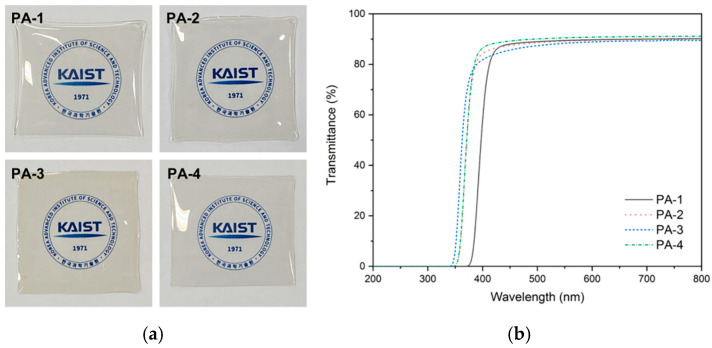
Images (**a**) and UV-vis spectra (**b**) of the PA films.

Tough films of the PAs were obtained by solution-casting from their DMAc solution. As shown in Figure 7, due to the lack of crystallinity, all the polymer films were colorless and highly transparent. The bulky, electron-withdrawing CF_3_ pendant groups effectively suppress the color-inducing interchain charge transfer (CT) interactions among polymer main chains through inductive effect, steric hindrance, and diminished intermolecular cohesive force through the low polarizability of the carbon–fluorine bond. Moreover, irregularly placed CF_3_ groups further disrupt the CT interaction by frustrating the ordered packing of polymer chains as previously discussed in Figure 4 and Figure 5. As a consequence, unsymmetrical insertion of two trifluoromethyl groups onto polymer main chains effectively enhanced the optical properties of PAs.

Optical properties of the PA films were characterized by UV-vis spectrometry and the results are shown in Table 4. All the PAs showed high optical transparency throughout the visible wavelength region, and the PAs containing meta- or flexible linkage showed excellent light transparencies over 80% at both 400 nm and 550 nm. While para-linked **PA-1** also exhibited a high transparency of 89% at 550 nm, it showed slightly less transparency at 400 nm than the other PAs, presumably owing to its relatively higher degree of chain alignment. **PA-1** also showed a longer cutoff wavelength (368 nm) than the other PAs (~340 nm) for the same reason. On the other hand, **PA-4** exhibited the best result because of the bulky, twisted hexafluoroisopropylidene bridge and its light transparency exceeds 90% at 550 nm. The color intensity of the PA films was evaluated quantitatively via colorimetry. The yellow index (YI) value of the PA films varies from 1.8–3.6 depending on diacid structure and decreased in the following order: **PA-3** > **PA-1** > **PA-2** > **PA-4**. Particularly, all the PAs except **PA-3** exhibited a very low YI value under 3, and the most colorless **PA-4** had the lowest YI value of 1.8. Given the fact that a YI value under 3 is often regarded as a technical threshold for transparent display [46], these colorless PA films could be suitable polymeric materials for technical application.

When compared to the PIs derived from the same diamine monomer [40], the PAs described here clearly showed less coloration and better optical properties. For instance, **PA-1** and **PA-4** have higher light transparencies at 400 nm as compared to its analogous PIs from pyromellitic dianhydride (*T*_400_ = 3%) and 4,4′-hexafluoroisopropylidenediphthalic anhydride (*T*_400_ = 57%) because of the absence of crystallinity. The complete replacement of electron-deficient dianhydride unit of PIs into diacid effectively alleviates the interchain CT interactions and results in reduced coloration of the polymer films. Taking into account that the PAs still retain high *T*_g_ over 300 °C, this result suggests that fluorinated PAs could also be a reliable option for achieving transparent polymeric material with a high glass transition temperature.

The refractive indices and birefringences of the PA films were obtained by using a prism-coupling method equipped with laser beams (633 nm and 1310 nm). As shown in Table 5, the entire polymers exhibited low refractive indices (n_av_) around 1.5333–1.5833 at 633 nm, and 1.5115–1.5682 at 1310 nm. The low refractive indices of the PAs stem from the weak molecular polarizability together with low density of the polymer films presumably caused by unsymmetrical incorporation of two CF_3_ groups. Because of the high fluorine content, **PA-4** exhibited much less n_av_ value than the other PAs. The birefringence values (Δn) of the PAs calculated from the difference of n_TE_ and n_TM_ were in the range of 0.0332–0.0628 at 633 nm, and 0.0295–0.0553 at 1310 nm. **PA-1** exhibited the highest birefringence among the PAs prepared in this study mainly because the high chain stiffness induced chain alignments parallel to the film x-y plane. In contrast, **PA-4** showed the lowest birefringence, suggesting that the segmental orientation as well as the linear polarizability of **PA-4** are the most isotropic among the PAs. The dielectric constant (ε) could be calculated from the refractive index by using Maxwell’s equation, ε ≈ n^2^. The ε value at 1 MHz was estimated to be ε ≈ 1.10 n_av_^2^, including approximately a 10% contribution owing to the infrared absorption [47]. The dielectric constants of the PA films obtained from the average refractive indices were in the range of 2.59–2.80 at 633 nm, and 2.51–2.71 at 1310 nm. The low dielectric constants of PAs could be attributed to the introduction of low polarizable trifluoromethyl groups on the polymer chains.

## 4. Conclusions

Unsymmetrically fluorinated polyamides were synthesized from the unsymmetrical diamine monomer having CF_3_ groups. In accordance with our previous results on fluorinated high- performance polymers, unsymmetrical attachment of trifluoromethyl groups onto polymer main chains was effective for aromatic polyamides in improving their optical properties as well as their solubility without compromising their good thermal stability. The soluble aromatic PAs can be readily processed into a flexible freestanding film via solution-casting and all the obtained polymer films were highly transparent and colorless. Moreover, all PA films had a high glass transition temperature over 300 °C owing to the presence of two bulky trifluoromethyl groups at *ortho*-positions of aryl ether bond. The transparent PA films with a high glass transition temperature are expected to find useful applications in flexible electronic devices.

## Figures and Tables

**Table 1:** Characterization of the synthesized PAs.

Polymer Code	η_inh_ (dL/g) ^a^	*M*_n_ (kDa) ^b^	*M*_w_ (kDa) ^b^	*M*_w_/*M*_n_ ^b^	Formula of PA (Formula Weight)		C	H	N
**P** **A-1**	1.28	61.8	128.4	2.07	(C_22_H_12_F_6_N_2_O_3_)_n_ (466.33)_n_	Calcd Found	56.66 54.78	2.59 2.77	6.01 5.78
**P** **A-2**	0.80	55.0	133.0	2.42	(C_22_H_12_F_6_N_2_O_3_)_n_ (466.33)_n_	Calcd Found	56.66 55.43	2.59 2.77	6.01 5.83
**P** **A-3**	1.06	45.4	119.0	2.62	(C_28_H_1__6_F_6_N_2_O_4_)_n_ (558.43)_n_	Calcd Found	60.22 58.50	2.89 3.00	5.02 4.73
**P** **A-** **4**	0.81	51.8	119.7	2.31	(C_31_H_16_F_12_N_2_O_3_)_n_ (692.45)_n_	Calcd Found	53.77 52.77	2.33 2.46	4.05 3.84

^a^ Measured in DMAc (0.5 g/dL) at 30 °C. ^b^ Determined by GPC in THF at 35 °C (polystyrene standards).

**Table 2:** Solubility of the Pas ^a^.

Solvents ^b^	PA-1	PA-2	PA-3	PA-4
NMP	++	++	++	++
DMAc	++	++	++	++
DMF	++	++	++	++
DMSO	++	++	++	+
*m*-cresol	++	++	++	++
THF	++	++	++	++
Acetone	-s	-s	-s	++
Ethyl acetate	-	-	-	-s
Anisole	-	-	-	-
Chloroform	-	-	-	-
Acetonitrile	-	-	-	-

^a^ Solubility was determined with 10 mg of polymer in 1 mL of solvent. ++, soluble at room temperature; +, soluble on heating; -s, swelling; -, insoluble. ^b^ Abbreviations: NMP (*N*-methyl-2-pyrrolidone); DMAc (*N*,*N*-dimethylacetamide); DMF (*N*,*N*-dimethylformamide); DMSO (dimethyl sulfoxide); THF (tetrahydrofuran).

**Table 3:** Thermal properties of the PAs.

Polymer Code	*T*_d5_ (°C) ^a^		*T*_g_ (°C) ^b^	CTE (ppm/°C) ^c^		Char Yield (%) ^d^
	In N_2_	In Air		2nd Run	3rd Run	
**PA-1**	441	437	334	49.8	49.7	50
**PA-2**	437	434	310	58.1	58.0	52
**PA-3**	440	427	308	57.9	57.9	46
**PA-4**	465	458	311	76.1	76.7	47

^a^ 5% weight loss temperature, obtained by TGA at a heating rate of 10 °C/min. ^b^ Measured by DSC (second scan) in N_2_ at a heating rate of 10 °C/min. ^c^ Linear coefficient of thermal expansion, measured by TMA in N_2_ at a heating rate of 5 °C/min. ^d^ Residual weight at 800 °C in nitrogen.

**Table 4:** Optical properties of the PAs.

Polymer Code	Cutoff Wavelength (nm)	Transmittance		YI ^a^	Film Thickness (μm)
		At 400 nm	At 550 nm		
**PA-1**	368	58.4	89.4	2.5	~30
**PA-2**	344	84.3	89.2	2.3	~30
**PA-3**	337	81.8	88.4	3.6	~30
**PA-4**	343	86.5	90.5	1.8	~30

^a^ Yellow index was calculated according to ASTM D1925 formula.

**Table 5:** Refractive indices of the PAs.

λ (nm)	Polymer Code	*n*_TE_^a^	*n*_TM_^b^	*n*_av_^c^	Δ*n* ^d^	ε ^e^	d (μm) ^f^
633	**PA-1**	1.6042	1.5414	1.5833	0.0628	2.76	6.8
	**PA-2**	1.5901	1.5534	1.5779	0.0367	2.74	8.8
	**PA-3**	1.6122	1.5628	1.5957	0.0494	2.80	4.4
	**PA-4**	1.5444	1.5112	1.5333	0.0332	2.59	8.4
1310	**PA-1**	1.5743	1.5190	1.5559	0.0553	2.66	6.7
	**PA-2**	1.5625	1.5298	1.5516	0.0327	2.65	8.8
	**PA-3**	1.5830	1.5387	1.5682	0.0443	2.71	4.3
	**PA-4**	1.5213	1.4918	1.5115	0.0295	2.51	8.0

^a^*n*_TE_: the in-plane refractive index. ^b^*n*_TM_: the out-of plane refractive index. ^c^*n*_av_: the average refractive index (*n*_av_ = (2*n*_TE_ + *n*_TM_)/3). ^d^ Δ*n*: birefringence (*n*_TE_ − *n*_TM_). ^e^ Dielectric constant estimated from the refractive index: ε ≈ 1.10 *n*_av_^2^. ^f^ Film thickness for the refractive index measured.

## Data Availability

Not applicable.

## Supplementary Materials

The following are available online at https://www.mdpi.com/article/10.3390/polym14030501/s1, Figure S1: GPC diagrams of PA-1, PA-2, PA-3 and PA-4., Figure S2: ^13^C NMR spectrum of PA-1., Figure S3: ^13^C NMR spectrum of PA-2., Figure S4: ^13^C NMR spectrum of PA-3., Figure S5: ^13^C NMR spectrum of PA-4.

## References

[1] Choi M.-C., Kim Y., Ha C.-S. (2008). Polymers for flexible displays: From material selection to device applications. Prog. Polym. Sci..

[2] Logothetidis S. (2008). Flexible organic electronic devices: Materials, process and applications. Mater. Sci. Eng. B.

[3] Lim H., Cho W.J., Ha C.S., Ando S., Kim Y.K., Park C.H., Lee K. (2002). Flexible organic electroluminescent devices based on fluorine-containing colorless polyimide substrates. Adv. Mater..

[4] Yan M., Kim T.W., Erlat A.G., Pellow M., Foust D.F., Liu J., Schaepkens M., Heller C.M., Mcconnelee P.A., Feist T.P. (2005). A transparent, high barrier, and high heat substrate for organic electronics. Proc. IEEE.

[5] Nakano S., Saito N., Miura K., Sakano T., Ueda T., Sugi K., Yamaguchi H., Amemiya I., Hiramatsu M., Ishida A. (2012). Highly reliable a-IGZO TFTs on a plastic substrate for flexible AMOLED displays. J. Soc. Inf. Disp..

[6] Zhu H., Shin E.S., Liu A., Ji D., Xu Y., Noh Y.Y. (2020). Printable Semiconductors for Backplane TFTs of Flexible OLED Displays. Adv. Funct. Mater..

[7] Yu M.C., Ruan D.B., Liu P.T., Chien T.C., Chiu Y.C., Gan K.J., Sze S.M. (2020). High Performance Transparent a-IGZO Thin Film Transistors with ALD-HfO2Gate Insulator on Colorless Polyimide Substrate. IEEE Trans. Nanotechnol..

[8] MacDonald W.A. (2004). Engineered films for display technologies. J. Mater. Chem..

[9] Yi L., Huang W., Yan D. (2017). Polyimides with side groups: Synthesis and effects of side groups on their properties. J. Polym. Sci. Part A Polym. Chem..

[10] Tapaswi P.K., Ha C.-S. (2019). Recent Trends on Transparent Colorless Polyimides with Balanced Thermal and Optical Properties: Design and Synthesis. Macromol. Chem. Phys..

[11] Liaw D.-J., Wang K.-L., Huang Y.-C., Lee K.-R., Lai J.-Y., Ha C.-S. (2012). Advanced polyimide materials: Syntheses, physical properties and applications. Prog. Polym. Sci..

[12] Hasegawa M., Horie K. (2001). Photophysics, photochemistry, and optical properties of polyimides. Prog. Polym. Sci..

[13] Chern Y.T., Tsai J.Y. (2008). Low dielectric constant and high organosolubility of novel polyimide derived from unsymmetric l,4-Bis(4-aminophenoxy)-2,6-di-tert-butylbenzene. Macromolecules.

[14] Bong S., Yeo H., Goh M., Ku B.C., Kim Y.Y., Bong P.H., Park B., You N.H. (2016). Synthesis and characterization of colorless polyimides derived from 4-(4-aminophenoxy)-2,6-dimethylaniline. Macromol. Res..

[15] Yang C.-P., Hsiao S.-H., Yang H.-W. (2000). Organosoluble optically transparent poly(ether imide)s based on atert-butylhydroquinone bis(ether anhydride). Macromol. Chem. Phys..

[16] Dhara M.G., Banerjee S. (2010). Fluorinated high-performance polymers: Poly(arylene ether)s and aromatic polyimides containing trifluoromethyl groups. Prog. Polym. Sci..

[17] Hasegawa M., Ishigami T., Ishii J., Sugiura K., Fujii M. (2013). Solution-processable transparent polyimides with low coefficients of thermal expansion and self-orientation behavior induced by solution casting. Eur. Polym. J..

[18] Hasegawa M., Watanabe Y., Tsukuda S., Ishii J. (2016). Solution-processable colorless polyimides with ultralow coefficients of thermal expansion for optoelectronic applications. Polym. Int..

[19] Reglero Ruiz J.A., Trigo-López M., García F.C., García J.M. (2017). Functional aromatic polyamides. Polymers.

[20] Yin J., Mao D., Fan B. (2021). Copolyamide-Imide Membrane with Low CTE and CME for Potential Space Optical Applications. Polymers.

[21] In I., Kim S.Y. (2006). Soluble wholly aromatic polyamides containing unsymmetrical pyridyl ether linkages. Polymer.

[22] Hsiao S.H., Lin K.H. (2004). Soluble aromatic polyamides bearing asymmetrical diaryl ether groups. Polymer.

[23] Lee B., Byun T., Kim S.D., Kang H.A., Kim S.Y., Chung I.S. (2015). Soluble para-linked aromatic polyamides with pendent groups. Macromol. Res..

[24] Sheng S.R., Pei X.L., Huang Z.Z., Liu X.L., Song C.S. (2009). Novel soluble fluorinated aromatic polyamides derived from 2-(4-trifluoromethylphenoxy)terephthaloyl chloride with various aromatic diamines. Eur. Polym. J..

[25] Behniafar H., Khosravi-borna S. (2009). Synthesis and characterization of novel aromatic polyamides derived from 2, 2′-bis(p-phenoxyphenyl)-4, 4′-diaminodiphenyl ether. Polym. Int..

[26] Sheng S.R., Ma C.X., Jiang J.W., Huang Z.Z., Song C.S. (2010). Synthesis and properties of novel aromatic polyamides with xanthene cardo groups. J. Appl. Polym. Sci..

[27] Liou G.-S., Maruyama M., Kakimoto M.-A., Imai Y. (1993). Preparation and properties of aromatic polyamides from 2,2′-bis(p-aminophenoxy) biphenyl or 2,2′-bis(p-aminophenoxy)-1,1′-binaphthyl and aromatic dicarboxylic acids. J. Polym. Sci. Part A Polym. Chem..

[28] Banerjee S., William A. (2015). Handbook of Specialty Fluorinated Polymers.

[29] Rogers H.G., Gaudiana R.A., Hollinsed W.C., Kalyanaraman P.S., Manello J.S., Mcgowan C., Minns R.A., Sahatjian R. (1985). Highly Amorphous, Birefringent, Para-Linked Aromatic Polyamides. Macromolecules.

[30] Gaudiana R.A., Minns R.A., Rogers H.G., Sinta R., Taylor L.D., Kalyanaraman P., McGowan C. (1987). Molecular factors affecting solubility in rigid-rod polyamides. J. Polym. Sci. Part A Polym. Chem..

[31] Takada K., Mae Y., Kaneko T. (2018). Fluorinated and bio-based polyamides with high transparencies and low yellowness index. Polymers.

[32] Murad A.R., Iraqi A., Aziz S.B., Hi H., Abdullah S.N., Brza M.A., Abdulwahid R.T. (2020). Influence of Fluorine Substitution on the Optical, Thermal, Electrochemical and Structural Properties of Carbazole-Benzothiadiazole Dicarboxylic Imide Alternate Copolymers. Polymers.

[33] Kim S.D., Lee B., Byun T., Chung I.S., Park J., Shin I., Ahn N.Y., Seo M., Lee Y., Kim Y. (2018). Poly(amide-imide) materials for transparent and flexible displays. Sci. Adv..

[34] Matsuura T., Hasuda Y., Nishi S., Yamada N. (1991). Polyimide derived from 2,2′-bis(trifluoromethyl)-4,4′-diaminobiphenyl. 1. Synthesis and characterization of polyimides prepared with 2,2′-bis(3,4-dicarboxyphenyl)hexafluoropropane dianhydride or pyromellitic dianhydride. Macromolecules.

[35] Ma C.X., Sheng S.R., Wei M.H., He W., Song C.S. (2010). High-optical transparency and low-dielectric constant of new organosoluble polyamides containing trifluoromethyl and xanthene groups. J. Appl. Polym. Sci..

[36] Wang C.Y., Li P.H., Li G., Jiang J.M. (2009). High optical transparency and low dielectric constant of novel organosoluble poly(ether ketone amide)s derived from an unsymmetrical diamine containing trifluoromethyl and methyl pendant groups. Colloid Polym. Sci..

[37] Liaw D.J. (2005). Optically high transparency and light color of organosoluble polyamides containing trifluoromethyl and kink diphenylmethylene linkage. J. Polym. Sci. Part A Polym. Chem..

[38] Liaw D.J., Huang C.C., Chen W.H. (2006). Color lightness and highly organosoluble fluorinated polyamides, polyimides and poly(amide-imide)s based on noncoplanar 2,2′-dimethyl-4,4′- biphenylene units. Polymer.

[39] Li P., Wang C., Li G., Jiang J. (2010). High optical transparency and low dielectric constant of organosoluble poly(aryl ether amides) based on 1, 2-bis (4-amino-2-trifluoromethylphenoxy) benzene and aromatic dicarboxylic acids. J. Macromol. Sci. Part A Pure Appl. Chem..

[40] Kim S.D., Kim S.Y., Chung I.S. (2013). Soluble and transparent polyimides from unsymmetrical diamine containing two trifluoromethyl groups. J. Polym. Sci. Part A Polym. Chem..

[41] Yamazaki N., Higashi F., Kawabata J. (1974). Studies on reactions of the N-phosphonium salts of pyridines. XI. Preparation of polypeptides and polyamides by means of triaryl phosphites in pyridine. J. Polym. Sci. Polym. Chem. Ed..

[42] Zhang W.Q., Wang X.L., Liu G.C., Chen L., Wang Y.Z. (2016). Thermal transition behaviors, solubility, and mechanical properties of wholly aromatic para-, meta -poly(ether-amide)s: Effect on numbers of para -aryl ether linkages. RSC Adv..

[43] Yang C.P., Chen R.S., Chen K.H., Chen Y.P. (2002). Synthesis and properties of novel fluorinated polyamides based on 2-trifluoromethyl-4,4′-diaminodiphenylether. J. Chinese Chem. Soc..

[44] Lee S., Jeong R., Seo M., Lee H.-S. (2020). Double-activated nucleophilic aromatic substitution polymerization by bis-ortho-trifluoromethyl groups to soluble para-poly(biphenylene oxide). Polymer.

[45] Ando S., Sekiguchi K., Mizoroki M., Okada T., Ishige R. (2018). Anisotropic Linear and Volumetric Thermal-Expansion Behaviors of Self-Standing Polyimide Films Analyzed by Thermomechanical Analysis (TMA) and Optical Interferometry. Macromol. Chem. Phys..

[46] Hasegawa M. (2017). Development of Solution-Processable, Optically Transparent Polyimides with Ultra-Low Linear Coefficients of Thermal Expansion. Polymers.

[47] Wooten F. (1972). Optical Properties of Solids.

