# Supplementary material for: Highly Transparent Aromatic Polyamides from Unsymmetrical Diamine with Trifluoromethyl Groups
Source: Polymers (Basel). 2022 Jan 27;14(3):501. doi: 10.3390/polym14030501 (PMC8840359; doi:10.3390/polym14030501)
Supplement: Supplementary file 1 [file polymers-14-00501-s001.zip › polymers-1571791-supplementary.pdf]

Supplementary Materials

Highly Transparent Aromatic Polyamides from Unsymmetrical Diamine with Trifluoromethyl Groups

Seong Jong Kim‡, Inah Kang‡, Taejoon Byun, Jongho So, and Sang Youl Kim*

Department of Chemistry, Korea Advanced Institute of Science and Technology (KAIST), Daejeon 34141, Korea; krisj@kaist.ac.kr (S.J.K.); ruminaire@kaist.ac.kr (T.B.); jonghoso@kaist.ac.kr (J. S.)

* Correspondence: kimsy@kaist.ac.kr; Tel.: +82-42-350-2834

‡ These authors contributed equally

Supplementary Figures

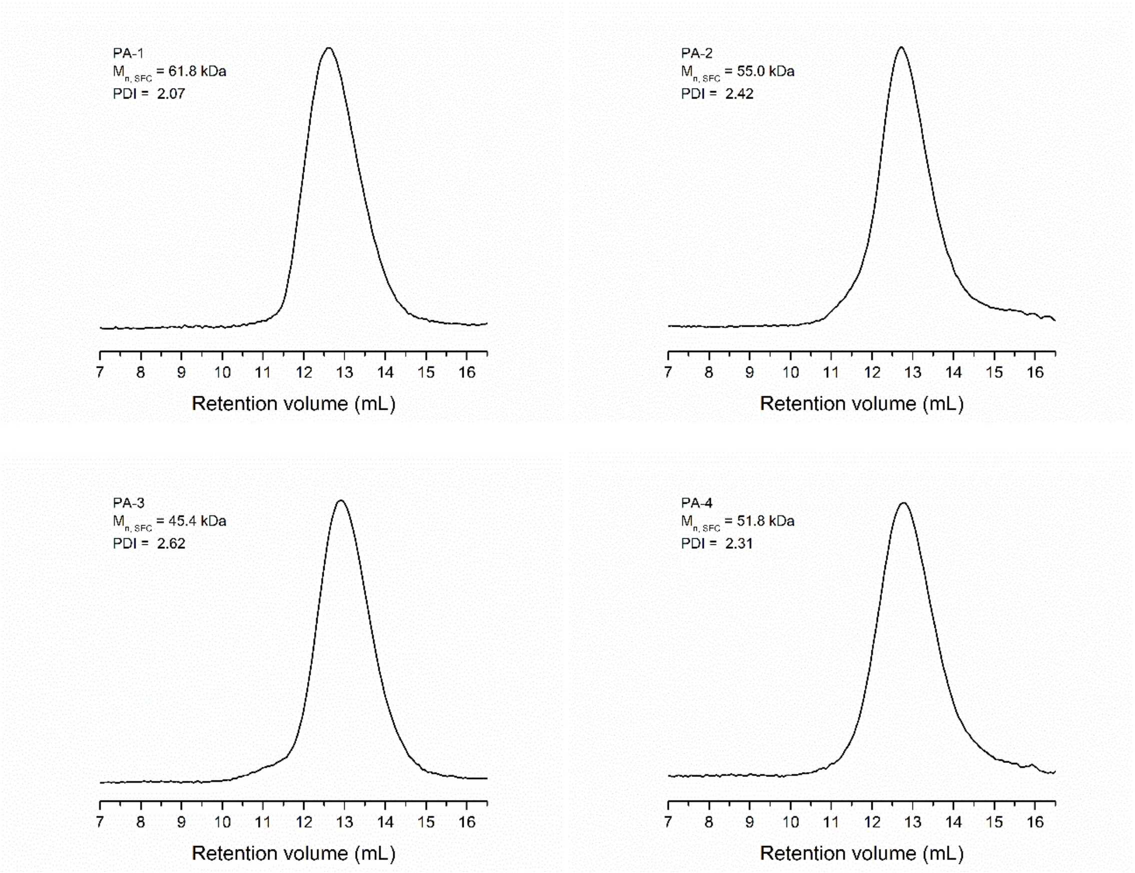

Figure S1. GPC diagrams of **PA-1, PA-2** , **PA-3** and **PA-4**.

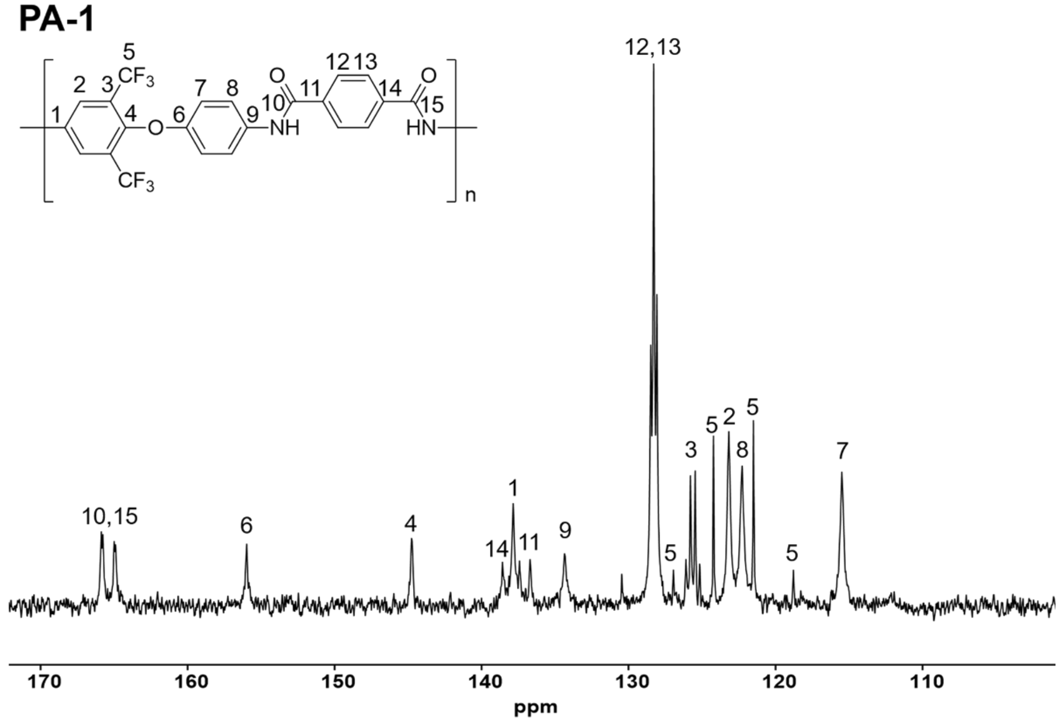

Figure S2. ^{13}C NMR spectrum of **PA-1**

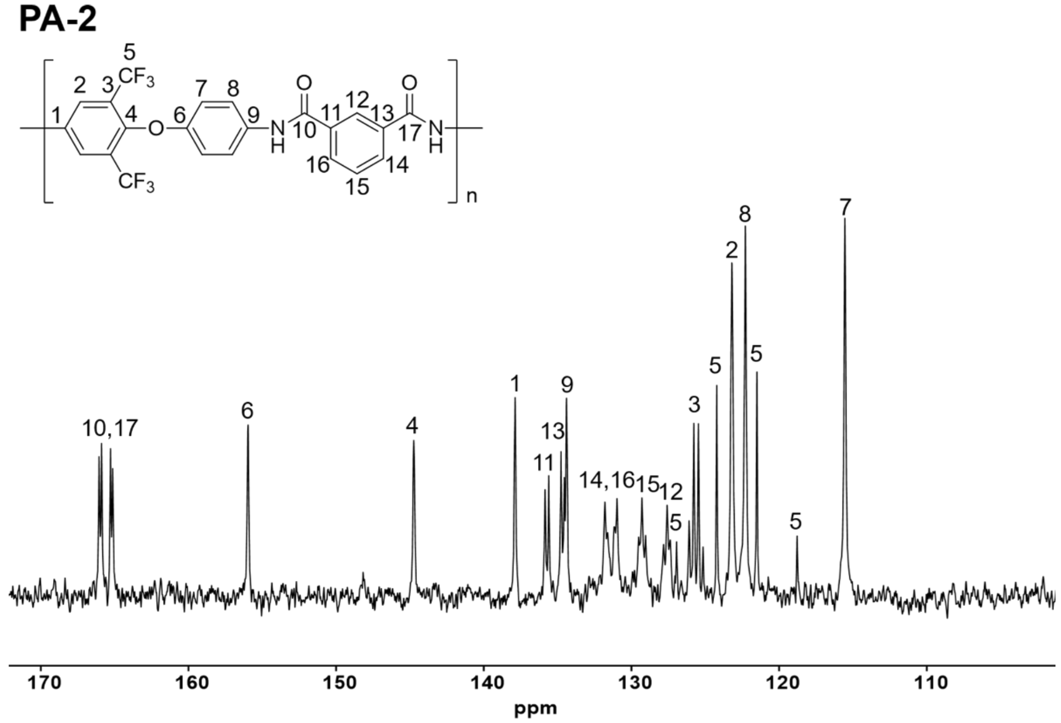

Figure S3. ^{13}C NMR spectrum of **PA-2**

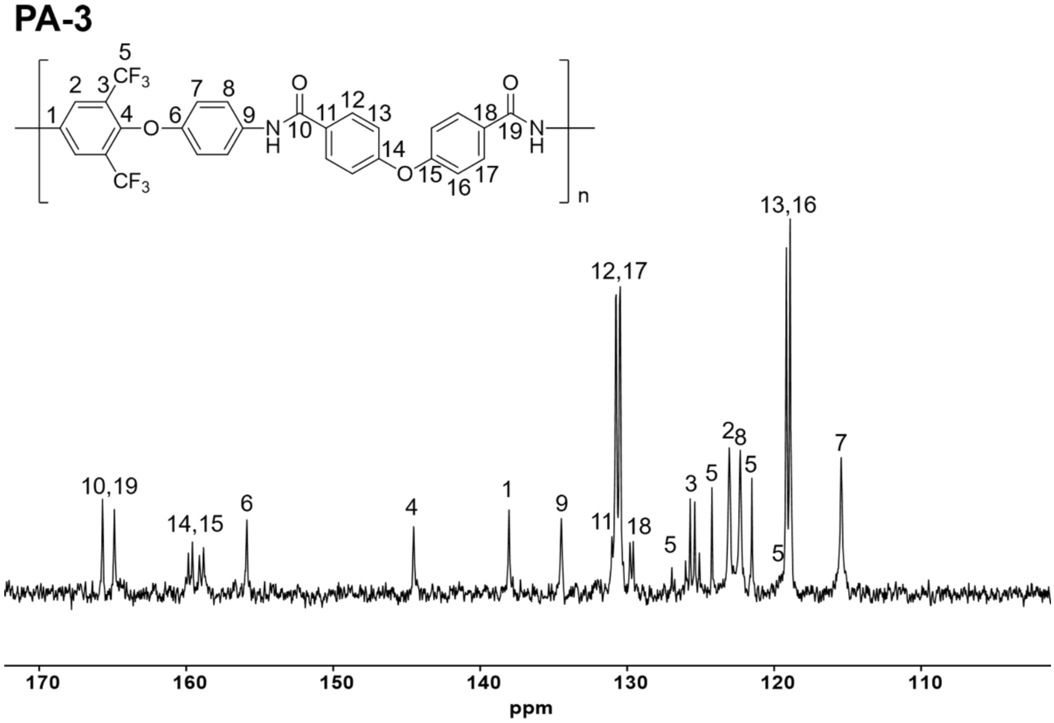

Figure S4. ^{13}C NMR spectrum of **PA-3**

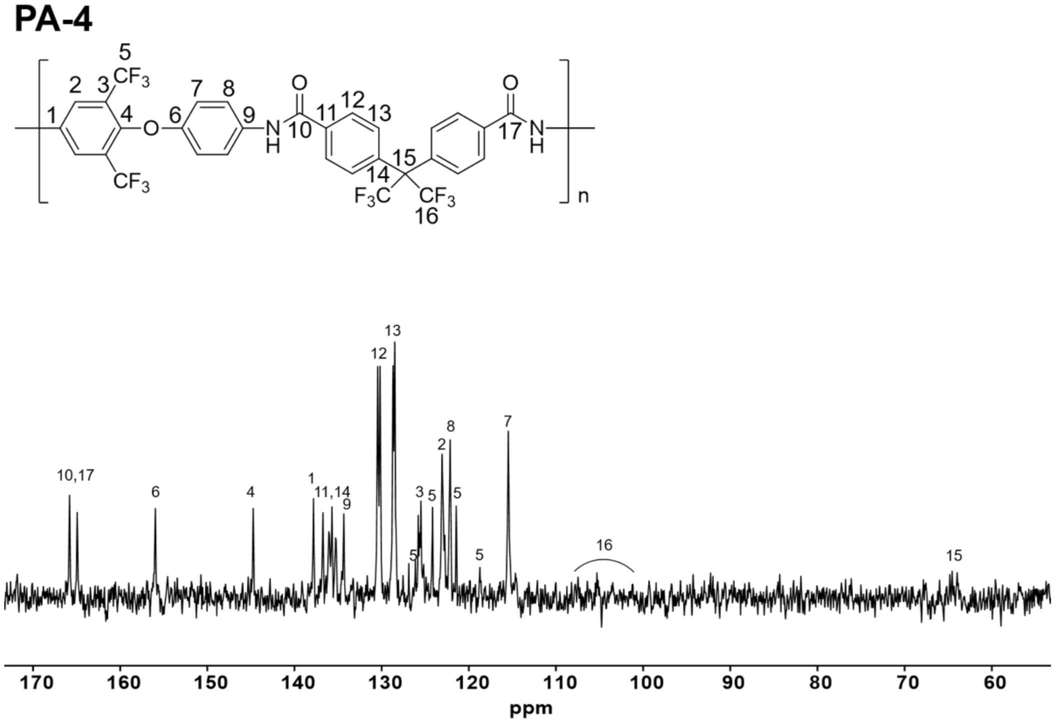

Figure S5. ^{13}C NMR spectrum of **PA-4**
